# The Common Fruit-Piercing Moth in the Pacific Region: A Survey of the Current State of a Significant Worldwide Economic Pest, *Eudocima phalonia* (Lepidoptera: Erebidae), with a Focus on New Caledonia

**DOI:** 10.3390/insects12020117

**Published:** 2021-01-29

**Authors:** Lise Leroy, Christian Mille, Bruno Fogliani

**Affiliations:** 1 Équipe ARBOREAL: “AgricultuRe BiOdiveRsité Et vALorisation”, Laboratoire d’Entomologie Appliquée, Station de Recherches Fruitières de Pocquereux, IAC, Institut Agronomique néo-Calédonien, P.O. Box 32, 98880 La Foa, New Caledonia; bruno.fogliani@unc.nc; 2 ISEA: Institut des Sciences Exactes et Appliquées, Universiteé de la Nouvelle-Calédonie, BP R4, 98851 Nouméa CEDEX, New Caledonia

**Keywords:** fruit-piercing moths, New Caledonia, integrated pest management

## Abstract

**Simple Summary:**

Fruit-piercing moths have long been cited as important pests in tropical and subtropical countries but genus as Eudocima, has recently gained in significance, and more specifically Eudocima phalonia (Linneaus). An overview of the current pest control proposed in the literature pointed the lack of sustainable integrated pest management. A synthesis of available data opens the research per-spectives that need to be encouraged in the ecological transition of our agricultural models.

**Abstract:**

When referring to fruit-piercing moths, the genus *Eudocima*, and more specifically *Eudocima phalonia* (Linneaus)*,* is cited as a worldwide crop pest. Damages associated with this pest are substantial on more than 100 fruit species, wherever it is encountered. In New Caledonia, the once occasional pest has become a serious threat to the current fruit arboriculture. Particularly devastating during outbreak periods, it has become an urgent need to find a suitable solution able to support farmers in the ecological transition of our agricultural models. This review proposes a synthesis of the existing data and publications on *E. phalonia*, worldwide and especially in New Caledonia, with recent observations. The assessment of this knowledge and the dynamics of the species in the territory of New Caledonia provide key information for a better prospect of adapted solutions.

## 1. Introduction

The majority of Lepidopteran crop pest species are economically important due to phytophagous larvae [1,2,3,4,5], but with fruit-piercing moths, it is the adults which cause significant damages to fruit crops. A recent review on Lepidoptera pest injury guilds rightly considered the extent of damages made by fruit-piercing moths and pointed out a pest status similar to highly polyphagous Lepidoptera pest larvae [6]. If their impacts are high, it is because they pierce the skin of fruit close to maturity, extract the nutritious juice and facilitate access by other secondary organisms which feed on damaged fruits, such as fungi, bacteria and other insects [7,8]. It is then necessary to distinguish primary and secondary fruit-piercers from other sucking moths which are not able to pierce the skin of a fruit. Depending on the characteristics of the fruit skin and the morphology of the moth proboscis, several authors have distinguished three categories in primary piercing moths: moths able to pierce a soft and thin-skinned fruit (i.e., tomato), a soft and thick-skinned fruit (i.e., guava) and a hard and thick-skinned fruit (i.e., orange) [9,10]. Only moths with a strong and sclerotized proboscis with erectile barbs, hooks and rasping spines are characterized as primary fruit-piercers of hard-skinned fruits and by extension, they are almost able to pierce any fruit skin type [9,10,11,12,13]. Even if the number of species able to pierce fruits is relatively large, only 20 species can have a significant economic impact in the world [14,15]. Among these twenty species, the genus *Eudocima* is largely represented as an important economic group with pest species such as *Eudocima phalonia* (L.), which has long been known as *E. fullonia* (Clerck), *Othreis fullonia* (Clerck), *Ophideres fullonica* (L.), one of the most damaging and widespread species in the southern hemisphere except for the Americas, where other species are recorded.

In this article, we review the biology of this fruit-piercing moth species and its economic importance in the world, with a focus on New Caledonia, where the *E. phalonia* problem has become considerably more acute over the last several years. During a normal year, damages to fruit crops caused by this moth are less than 30% (economic threshold) but the species can be highly destructive, up to 100%, when outbreaks occur. This outbreak phenomenon was first described as occurring every five years [16] but the extension of fruit crops, environmental changes, rising temperatures as well as reduced rainfalls have contributed to the imbalance of the population dynamic, which has led to annual outbreaks. Indeed, due to their short generation time and high reproductive rate, insects are more sensitive to climate variations but are also able to adapt quickly to these environmental changes [17]. While some methods of pest management are used to prevent high damages, their effectiveness is a matter of debate and accentuates the lack of a long-term sustainable solution. This is why a better knowledge of the new dynamics of the species, especially in New Caledonia, will offer us some new perspectives on pest management, either to be established or to be developed.

## 2. Geographic Distribution

**Figure 1:**
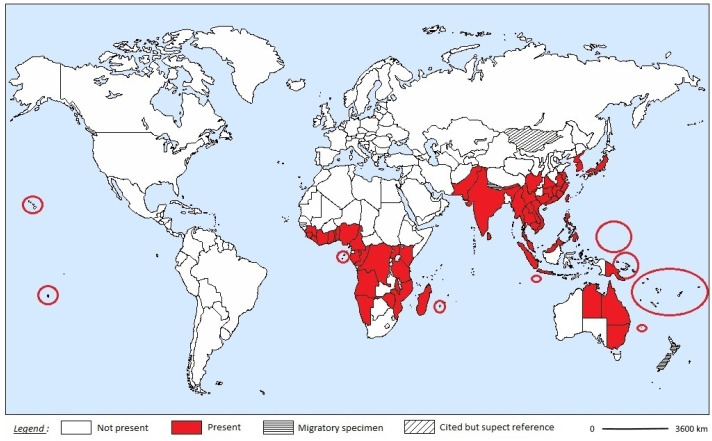
Distribution map of *Eudocima phalonia* based on available references.

*E. phalonia* is the most widespread species of *Eudocima*, found throughout the Pacific Region [16,18,19,20,21,22,23,24,25,26,27,28,29,30,31], in Southeast Asia, such as Thailand [8], Southeast China [32,33], India [9,34,35,36,37] and in several regions of Africa [7,38,39,40,41,42] (Figure 1). Its presence in Mongolia is considered suspect, because the climate is too different from other areas where *E. phalonia* has established some permanent populations [43]. In New Zealand, some isolated specimens have been caught [19]. In Nepal, specimens are observed at very high elevation such as 2650 and 3750 m [44]. Although no populations are maintained in these last three countries, these observations reflect the high migration capacity of this species. However, unlike Nepal and Mongolia, part of New Zealand’s land climate can be quite favorable to the development of *E. phalonia* and even if the main host plants (Menispermaceae) are not present in this territory, other larval hosts can be found in ornamental form [45]. Africa, Asia and Oceania are already colonized by *E. phalonia* but this is not the case for North and South America. Even if a number of *Eudocima* species are cited in the Americas, some authors rightly focus upon *E. phalonia* as a major potential threat and predict the distribution in climatically suitable areas to one third of the USA, mainly on the Eastern Coast [43]. This species has, however, been present in Hawaii for 25 years but no particular damage has been reported, mainly due to the action of several parasitoids [46].

## 3. *Eudocima phalonia* Complex Species

*Eudocima phalonia* can be classified as a paleotropical species. It is cited as native to the Indo-Malaysian Region, but recent works suppose a possible African origin, due to the presence of close relatives in the Afrotropics [47]. Indeed, close morphologies between *E. lequeuxi* (Brou and Zilli) found in Central Eastern and Southeastern Africa and the common *E. phalonia* make it even easier to confuse both species when they occur in sympatry [42]. Another species, *E. euryzona* (Hampson), endemic to Madagascar, shares the same features as the two previous species [48]. These two observations indicate an ancient occurrence of *E. phalonia* on the African mainland with a complex of species, where *E. phalonia* could be an ancestral form of both of them (*E. lequeuxi* and *E. euryzona*) or speciated from *E. lequeuxi* [49].

In the Pacific Region, historical data on *E. phalonia* presence indicate that the spreading started in the middle of the 19th century, probably with an ancestral form [47]. Among the 21 species of *Eudocima*, close morphologies are also observed between *E. phalonia* and two newly described species, *E. oliveri* (Zilli and Brou) in Vanuatu and *E. steppingstonia* (Brou, Klem, Zaspel and Zilli) in the Marquesas Islands [47]. Unfortunately, these two new species are described from specimens collected over the centuries, without recent observations. Despite geographical proximity with Vanuatu, *E. oliveri* has never been recorded from New Caledonia. This is also the case for the species *E. jordani* (Holland) [50]. Within this genus, three species are mentioned in New Caledonia, *E. materna* (L.), *E. salaminia* (Cramer) and *E. phalonia* [16,51]. However, due to proximity with Australia, it is not impossible to find a migrant specimen of *E. jordani* on the New Caledonian territory as some isolated specimens of *E. phalonia*, *E. paulii*, *E. salaminia* and *E. materna* have been caught in New Zealand from other Pacific islands. Nevertheless, this species has never been recorded from New Caledonia. In addition, the presence of *E. phalonia* local biotypes referenced in the Solomon Islands, Fiji or Samoa [47] does not exclude the possibility of a local one in New Caledonia, especially since the morphological characterization of New Caledonian populations has never been investigated. Furthermore, as is the case in many Pacific islands, the species develops on two larval host plant families (Menispermaceae and Fabaceae), contrary to its African counterpart, where this phenomenon is not recorded. This host plant adaptation may have been achieved through genetic changes because of ecological factors or environmental pressure, as can be seen in other insect models [52,53]. Molecular and phylogeographic analysis of these populations may help to track their past and current evolution along with genetic links and relationships with other species. If there is no doubt about the pest status of *E. phalonia*, it is advisable to investigate its life habits, population dynamics and occurrence of local biotypes, close relatives and other *Eudocima* species, in order to preserve species that are not problematic.

## 4. Economic Impact

High abundance of *Eudocima phalonia* populations is problematic for many fruit crops [7,27,28,32,37,39,54]. Nearly 100 fruit species (both cultivated and wild ones) are a potential source of food for this species [43]. Some fruits, such as mandarins, oranges, bananas, mangoes and tomatoes, are the preferred targets of the species [55,56,57,58]. Other fruits, such as lemons, grapefruits, peppers, melons, papayas and strawberries, are damaged during severe outbreaks. If the population density is too high and the optimal food resource is not available, moths can also attack immature green and acidic fruits [16,28,59,60,61]. However, both the abundance of this species and the extent of its damages (Table 1) are little quantified in the literature because they are often generically attributed to “fruit-piercing moths” in a broad sense, thus referring to different species which could potentially include *E. phalonia*.

In New Caledonia, adult moths can feed on more than 35 species of fruits, listed in Table 2, including recent observations. *Eudocima phalonia* was first cited in the territory in 1931 but the first attacks referred to a noctuid of the *Ophideres* genus may have been on coffee cherries in 1923 but the insect was not clearly identified [77]. The species was first classified as “an occasional pest in New Caledonia” because damages were usually around 4% on oranges and mandarins [14,78]. However, the incidence of the moth has since increased, especially with the expanded cultivation of *Citrus* species and their numerous cultivars. As a consequence, the species was ranked among the 10 species with a high economic importance [79] and as the fourth species of major economic importance in the Pacific Region [80]. This significance is essentially due to outbreak phenomena, when damages can rise to 100%. For example, in 1964 and 1969, 95% of oranges, 100% of tomatoes or 70–100% of fruits on the East Coast were damaged [14,55]. If orchards are close to the forest areas, 75–90% of the first citrus harvest can be lost [81].

In the Loyalty Islands and especially in Lifou and Maré, 75% of the fruits (mango, papaya and Malay apple) can be damaged during a severe attack [84]. In 2015, new attacks occurred and the citrus production was strongly affected, with 65% of the navel oranges, 50% of the mandarins (Ponkan cultivar), 76% of the early mandarins, 50% of grapefruits and 20–40% of oranges (Cadenara cultivar) and tangelos destroyed [93]. In 2020, on the main island so-called Grande Terre, damages were extremely significant in the coastal regions as in the valleys (60–100%) [96]. In 2016, 700 tons of citrus fruits were lost due to the moths, which represented up to 200 million Pacific Francs (around USD 1,903,000) in compensation paid to the farmers [97].

## 5. Larval Host Plants

*Eudocima phalonia* is the most generalist species, able to develop on a large number of plants of the family of the Menispermaceae (around 50 species) such as genera *Anamirta*, *Carronia*, *Cocculus*, *Dioscoreophyllum*, *Hypserpa*, *Legnephora*, *Pachygone*, *Pericampylus*, *Pleogyne*, *Stephania*, *Tiliacora*, *Tinospora* or *Triclisia*. In Asia, Africa and Australia, the pest develops only on representative genera of this family [13,24]. In Australia, there are 13 genera and 26 species, of which almost 60% are identified as larval hosts [98,99]. In Malaysia and Thailand, *Leea indica* (Vitaceae) is an alternative host plant for larval development [30,31].

In the Pacific Region, even if several taxa of the Menispermaceae are present (*Stephania japonica*, mainly, *S. hernandiaefolia*, *Tinospora homosepala*, *Pachygone ledermannii*, *P. vitiensis*, *Hypserpa ponapensis*, *Cocculus ferrandianus*), they are not strongly represented in the flora. Although the moth has preferential larval host plants, it is completely able to develop on secondary ones [27,28]. A remarkable fact is that, in the Pacific Region, Eudocima phalonia has expanded its range of larval host plants in Guam, Papua New Guinea, New Caledonia, Tonga, Hawai’i, French Polynesia, Vanuatu, Samoa, Fiji and Cook Islands, as it develops there on plants of the genus Erythrina (Fabaceae) [16,18,20,24,26,55,57,81,83,90,92,94,99,100,101,102].

In New Caledonia, the species is known from two *Stephania* taxa (*S. japonica* var japonica and *S. japonica* var timorensis) and several Fabaceae including five *Erythrina* (*Er. fusca*, *Er. subumbrans*, *Er. variegata*, *Er. variegata* var orientalis, *Er. variegata* var fastigiata) and *Vigna* sp. [15,16,55,77,90,92]. *Eudocima phalonia* has not been reported on *Tinospora* sp. and *Hypserpa* sp., although these two genera are present [103] with endemic representatives such as *Tinospora neocaledonica*, *Hypserpa neocaledonica* and *H. mackeii*. The prevalence of egg-laying appears to be greater on *Erythrina* species than on Menispermaceae [16], but this conclusion is probably biased by the abundance of *Erythrina* species in the environment. In addition, they do not share the same biotopes, and where Menispermecae are affected by decline and restricted to forest habitats, *Erythrina* species are quite widespread in both plains and forests, especially *Er. fusca.* The host shift on *Erythrina* can be explained by the low abundance of the Menispermaceae in some island countries [24,99]. This acceptance can be also explained by the fact that both botanical families share some chemical compounds with the tetracyclic *Erythrina*-type alkaloids [25]. If the larva of *Eudocima phalonia* does not survive on *Er. fusca* in India [13], in Australia, larvae can breed on *Er. variegata* only if they perform the first stage on *Tinospora smilacina* (Menispermaceae), as demonstrated in laboratory trials [104]. Indeed, if the first larval instar hatched comes from a fertilized egg directly oviposited on Menispermaceae, the first larval instar does not survive where it is transferred to the tenderest leaves of *Er. variegata*. However, when the first larval instar is deposited and hatched on *Er. variegata*, the larva develops normally when it is transferred to a Menispermaceae. This phenomenon could be related to Thorpe’s chemical theory [105]. If the egg is deposited directly on the surface of an *Erythrina* leaf, it absorbs the compounds emitted by the plant during the development of the embryo. When the larva hatches and consumes its chorion [16,88], it partly consumes similar compounds as the tetracyclic alkaloids. The adaptive capacity of *E. phalonia* would therefore be wider than the other representatives of the genus *Eudocima* and would therefore make it a dynamic species able to find several secondary hosts, making it easier for the species to maintain itself and support its survival [31].

It is also possible to find eggs on other plant families such as Poaceae (Gramineae), Zingiberaceae, Euphorbiaceae, Nyctaginaceae, Crassulaceae, Myrtaceae, Asteraceae (Compositae), Araliaceae or Fabaceae. In New Caledonia, 0.2% of fresh eggs can be found on more than 15 species often close to *Erythrina*. Even if the larva hatches, it does not survive on these plants [16].

## 6. Biology of the Species

The life cycle of *E. phalonia* is relatively well known and referenced by several authors, as shown in Table 3 and according to localities, it seems to show some variations. Climatic conditions and fruit availability can both strongly influence population abundance [8,30,31]. In most countries, the species is seasonal and populations are more abundant during the wet season [7,8,16,22,23,25,33,39,54,76,106,107]. The only cited exception is for Malaysia, where the species’ activity is greater during the dry season [30,31], but the concept of the dry season in an equatorial climate is relative. Outside the wet favorable season, moths are rarely observed [16,33] and larvae and imagines are very difficult to find [16,54]. However, moths can also be active on fruits later in the fruit production season [34,108].

The active period of the moth is situated in the first part of the night, from 7.30 pm to 12 pm, and declines after midnight, but moths can still be observed in the early morning until 5 am [9,39]. However, first arriving moths are more likely to be males, while females arrive later in orchards [54]. Nights with or without the moon do not seem to disturb moth activity, nor do fine or weak rains [9,16]. Although the presence of the moths can be positively correlated with the number of rainy days, minimum and maximum temperature and morning and evening relative humidity, this correlation is not systematic for all studied years [108]. In the literature, no diapause is recorded for this species (Table 3) and does not appear to have been observed since. However, *E. phalonia* nevertheless persist during unfavorable seasons and this implies the presence of at least one mechanism or behavior that helps the species to survive. If no diapause is listed, a quiescence phenomenon can be supposed due to a longer lifespan of each developmental stage during the cold season.

In New Caledonia, the optimal cycle has been described as 30 days during the warm and wet season (from November to April), compared to 54.5 days during the cool and dry season (from May to September) [16]. During the warm season, the egg stage lasts three to four days, followed by a larval stage lasting 16 days and a pupal stage between 11.5 and 14 days. During the cool season, lower ambient humidity and low temperatures increase the development time of eggs, larvae and pupae by 4 to 6 days, 21 to 29 days and 19 to 27 days, respectively. Consequently, during the warm and wet season, the life cycle of the fruit-piercing moth is 30 to 33 days, whereas it extends to 44 to 62 days in the cool season. In New Caledonia, *E. phalonia* arrives in orchards one hour after sunset, flies again after midnight and pierces the fruit at all hours [16]. During the night, there must be at least a phase during which the moths do not feed but fly away from the orchards in order to mate or to oviposit.

## 7. Predators and Parasitoids

### 7.1. Egg Parasitoids

Hymenoptera of the families of Encyrtidae, Eulophidae, Pteromalidae, Ichneumonidae and Braconidae comprise many species parasitizing the Lepidoptera [114]. It seems that the eggs and young larval instars are the essential targets of these parasitoids. Depending on the locality where they are studied and the oviposition behavior (solitary oviposition or in clusters) of *Ooencyrtus* sp., *Telenomus* sp. or *Trichogramma* sp., the species of these genera have variable efficacy. For example, in Guam, *Telenomus* sp. and *Ooencyrtus* sp. are more efficient on egg clusters of *Eudocima phalonia*, with 54.6% and 23.9% parasitism rates as opposed to solitary oviposited eggs. For *Trichogramma* sp., the parasitism rate is higher on solitary eggs [115]. In the Fiji Islands, *Telenomus* sp. is not as efficient and parasitizes less than 2% of eggs, whereas the parasitism rate of *Trichogramma* sp. ranges from two to 16%, depending on the species [59]. Other *Ooencyrtus* sp. or *Telenomus lucullus* (Nixon) (Hymenoptera, Encyrtidae and Scelionidae) can parasitize the eggs up to 95% [116]. In Hawai’i, the incidence of *Trichogramma ostriniae* (Pang & Chen) (Hymenoptera, Trichogrammatidae) is so effective that 95% of *E. phalonia* eggs are parasitized [117]. In addition, the introduction of *Trichogramma chilonis* (Ishii) (Hymenoptera, Trichogrammatidae) to control a Crambidae [46] has contributed to the population control of *E. phalonia* in this state.

Egg parasitoids in New Caledonia belong to *Ooencyrtus*, *Telenomus* and *Trichogramma* genera and their incidence varies between Micronesian islands [22]. *Ooencyrtus papilionis* (Ashmead) (Hymenoptera, Encyrtidae) is the main parasitoid of *E. phalonia* eggs and its parasitism rate shows an average of 30% in the mountains and 40% in the plains [16]. This species can parasitize between 20% and 55% on solitary eggs and 26.8% on egg clusters [16,81]. In captivity, a single female can parasitize up to 61 eggs [81] and, on average, two parasitoids are obtained per parasitized egg. Even if the wasp is not present all year round because of annual fluctuations, their variations follow the fluctuations of *E. phalonia* [22,81,90]. During the cool season and at the beginning of the warm one, parasitoids are less abundant in the environment, which also corresponds to the low abundance of moths and butterflies [81,90]. As a result, *Ooencyrtus papilionis* has secondary hosts as *Papilio amynthor* (Boisduval) or *P. montrouzieri* (Boisduval) (Lepidoptera, Papilionidae)*,* two butterflies of New Caledonia [16,81] of which the second one is endemic. These secondary hosts allow the maintenance of wasp populations in the environment. Other egg parasitoids, *Trichogramma chilonis* and *Telenomus lucullus*, are more rarely found, and their incidence is negligible in New Caledonia because of their small populations [16,81,90]. *Trichogramma chilonis* appears when populations of *E. phalonia* and *Ooencyrtus papilionis* are at their lowest [81]. Even if *Telenomus lucullus* is specialized on *E. phalonia* or other related species of the genus [116,118], its effect is especially low in New Caledonia [81].

### 7.2. Larval Parasitoids

Larval ecto- and endoparasites are mainly members of the Ichneumonidae, Eulophidae (Hymenoptera) and Tachinidae (Diptera) and, more rarely, Braconidae (Hymenoptera), Ceratopogonidae (Diptera) and some Mesostigmata mites. In Sri Lanka and India, *Apanteles* and other Braconidae species are able to parasitize *Eudocima phalonia* larvae [32]. In the Eulophidae family, the majority of parasitoids of the *Euplectrus* genus are found. In India, *Euplectrus maternus* (Bhatnagar) (Hymenoptera, Eulophidae) parasitizes *E. materna*, *E. phalonia* and larvae of *E. homaena* (Hübner) (Lepidoptera, Erebidae) but these parasitoids seem to have a minor impact on moth populations in field conditions [56]. In Guam, the species did not persist in agroecosystems when the wasp was introduced [79]. In Australia, another *Euplectrus*, *Eu. melanocephalus* (Girault) (Hymenoptera, Eulophidae), parasitizes *E. phalonia* but also other species of *Eudocima* such as *E. aurantia* (Moore) *E. cocalus* (Cramer), *E. iridescens* (Lucas) and *E. jordani* [119]. Even if other native wasps are active and present good possibilities for biological pest control [120], they are unable to provide sufficient control of moth populations during the summer peak [121] and a fortiori during an outbreak phenomenon.

In New Caledonia, larval ectoparasites and endoparasites are species of the Ichneumonidae, Eulophidae (Hymenoptera) and Tachinidae (Diptera) families. If some of them are quite common on *E. phalonia* larvae in New Caledonia, such as *Euplectrus platyhypenae* (Howard) (Hymenoptera, Eulophidae), other species such as *Lissopimpla pacifica* (Morley) and *Echthromorpha agrestoria* (Swederus) (both Hymenoptera, Ichneumonidae) develop in pupae [81]. Rarely observed on the pest species, these larval parasitoids may presumably have a little impact on *E. phalonia*. Even if this was not investigated in the territory, their low parasitism rates could be explained by the fact that *E. phalonia* is not necessarily their main host [16], as they do not show high specificity. Moreover, current observations on wild larva suggest a higher rate of larval parasitoids during the fresh season than in the wet season [88].

Finally, the endoparasitoid tachinid fly, *Winthemia caledoniae* (Mesnil) (Diptera, Tachinidae), is the most important parasitoid on larvae of *E. phalonia* in New Caledonia. It sometimes attacks the fourth larval instar but it is mainly the fifth instar that is parasitized [16,19]. The parasitism rate is 25.46% and can even locally rise to 100% of the larvae at the end of the outbreak peak [16,81,90]. Three to five pupae can emerge from one parasitized *E. phalonia* pupae. During its lifetime, a female fly can lay a total of up to 80 eggs and 46% of eggs deposited on fifth instar larvae of *E. phalonia* give pupae [81]. However, the abundance of this tachinid fly is high at the end of the warm season, during the cool season and can be locally effective in small patches. Unfortunately, this parasitoid comes into action when moth populations decline [16], and recent observations show that the fly is not present in all biotopes and seems to be dispersed only in patches. We observed the same fact during *E. phalonia* larvae collection for a lab-rearing project [88]. This phenomenon is explained by the fact that *Winthemia caledoniae* pupae cannot survive in dry climates during the warm season and exploit several secondary hosts [16,81].

### 7.3. Predators

Regarding moths, bats are potential predators in Africa and in Micronesia [7]. Predation by Assassin Bugs (Reduviidae) ranges from 1% to 40% [22]. Nevertheless, most of the studies have investigated parasitoid impacts on *E. phalonia* for biological control issues. If predators need to be considered as an important factor of regulation, the potential for introducing predators is, on the other hand, more difficult because of their wide prey spectrum. In addition, observations of predators on *E. phalonia* are sparse in the literature, except in New Caledonia.

Predators in New Caledonia are well known [15,16,81,90]. Eggs, larvae, pupae and moths are the prey of a wide range of species, with different impacts due to the predator’s biology. For example, larvae of *Mallada noumeana* (Navás) (Neuroptera, Chrysopidae) are important egg predators when they are present in the environment and a chrysopid larva can consume more than seven eggs per day regardless of the egg’s age or if it has already been parasitized by wasps [16,81]. *Chrysoperla congrua* (Walker) and *Mallada basalis* (Walker) (Neuroptera, Chrysopidae), cited by the same author, are probably also some good predators of *E. phalonia* [122]. For these species, their density is high only after a large population of moths and reaches their maximum at the end of April (end of the warm season), when the moth density decreases. These predators are, however, themselves highly parasitized [81]. In contrast, Assassin Bugs of the genus *Ploiaria* as *P. glabella* (Wygodzinsky) (Hemiptera, Reduviidae) consume fewer eggs than chrysopids (three per day) and are occasional predators and prefer fresh eggs. For example, *Montrouzieriellus falleni* (Guérin-Méneville) (Hemiptera, Pentatomidae) is mainly found in the mountains, absent in the dry season and thus has minor importance in plains [81]. Other predators are cited but their impact is relatively low compared to the egg predators mentioned above. *Ooencyrtus papilionis*, mentioned in the previous subtitle, can also feed on two-day-old eggs. Before this predatory feeding, the parasitic behavior prevails. Ants are sometimes observed but their action remains localized and occasional; however, they can still attack an entire egg cluster [16,81,88]. Nutritionally, eggs are overall a very rich food source for predators, but in New Caledonia, the species identified as egg predators only accounts for 15% of egg losses [16,81].

Even if larval predators are less numerous than egg ones, they are equally generalist and opportunistic and do not especially feed on *E. phalonia* larvae. Moreover, this concerns only the third, fourth and fifth larval instars or pupae. Predation on freshly hatched larvae (1st instar) has been rarely observed but ants and birds can be identified as their main predators—in particular, *Acridotheres tristis* (L.) (Aves, Saturnidae) or the Myna bird, an invasive species, and *Rhipidura* spp. as *R. spilodera* ssp. * verreauxi* G.R. (Gray) (Aves, Rhipiduridae), an endemic bird which attacks older larvae (second and third larval instars) [90]. *Megalurulus mariei* (Verreaux) (Aves, Locustellidae) and *Zosterops* spp. or silver-eyed birds (Aves, Zosteropidae) are cited as larval predators but are much rarer [16]. If bird predation is rarely observed, regulation action on *E. phalonia* larvae is mainly visible during outbreaks [81,94]. The yellow paper wasp, *Polistes olivaceus* (de Geer) (Hymenoptera, Vespidae), accidentally introduced, is also one of the most important predators of larval and pupal stages [16]. A nest of 100 wasps can consume, for example, seven second-instar larvae, 15 third-instar larvae and 10 fourth-instar larvae of *E. phalonia* per day [16]. Even if it has not yet been investigated, *Polistes stigma townswillensis* (Giordani Soika) (Hymenoptera, Vespidae), a second species of Vespidae which arrived in New Caledonia during the mid-nineties, shows certainly the same predation ability [123]. All of these predators can have a particular hunting behavior during *E. phalonia* outbreaks and attack in groups. Even if larvae are not completely consumed, they are at least injured and subsequently succumb to these attacks [16,81]. However, this exceptional behavior is not sufficient to control moth outbreaks [16]. Although few predators on adults are mentioned, the white-breasted woodswallow (*Artamus leucorynchus* (L.) (Aves, Artamidae)) has been observed hunting moths at sunset or very early in the morning [88]. Bats and some gecko species are also susceptible to being moth predators but data are missing about their potential predation rate [88]. However, thanks to tympanic organs, avoidance behavior triggered by the perception of ultrasounds is observed with bats. It induces a moth to drop to the ground or engage in a circular and rapid flight in order to escape predators [124,125,126,127]. In addition, recent evidence indicates that perceived ultrasounds can adversely affect insect calling behavior and reproductive fitness [128,129,130]. It could be very interesting to test if ultrasounds can have such effects on *Eudocima phalonia* and if this strategy could be applied in combination with another, in a “push and pull” strategy.

## 8. Outbreak Phenomena in New Caledonia

The first outbreak phenomenon of *Eudocima phalonia* in New Caledonia was described in 1942 and happened in 1931 [77]. These outbreaks appear to occur, on average, every five years, can disappear for several consecutive years and reappear suddenly in some years [16]. Since then, numerous episodes have taken place in the territory, as in 1958, 1964 and 1969 [16,92], then in 1995, 1996, 2003, 2006 and 2007, until recently in 2015–2018 and 2020. Some outbreak data are certainly missing between 1969 and 1995. This phenomenon does not only take place in New Caledonia, since it has also been observed in other Pacific islands [118]. *E. phalonia* outbreaks are also cited to occur in Africa, [7] but due to the sympatric presence of *E. lequeuxi* and *E. phalonia*, it is possible that the phenomenon refers to one, the other or to both species. An outbreak is often the result of several factors, and in the case of *E. phalonia*, observations made by different authors suggest that four major determinants contribute to the phenomenon: (i) influence of seasonality (temperature/humidity/day length), (ii) availability of larval host plants and host fruits, (iii) ability of individuals to disperse or migrate, and (iv) the deficiency of the biocontrol agent complex. These factors are also intrinsically linked to each other because variations in temperature and humidity are modulated by seasonality, which influences food and the availability of the biocontrol agent complex.

### 8.1. Seasonal Influence

Seasonality is governed by three modalities that influence the development cycle of plant and animal communities: temperature, rain and humidity. Whether in Australia, India, Thailand, Africa, New Caledonia, Sri Lanka or Guam, the favorable period for the development of this species is a wet and a rainy season [7,8,16,32,33,38,39] and often coincides with the warm season as in New Caledonia. In addition, increasing or decreasing temperatures can modulate the period of activity [131] and the length of biological development of lepidopterous insects. In New Caledonia, cyclonic or excessive rains are not necessary to enhance moth population growth but moderate rains in spring may encourage the early arrival of moths in the plains. Once a dry period (at least six months) is followed by a delay in the rains, excessive or moderate rains lead to quick moth population growth. This scheme leads to outbreak phenomena in various moth species [2,15,132] including *E. phalonia* in New Caledonia [16,90]. This critical drought period runs from September to December (the year before the outbreak) and during this period, the deficit of water can have serious consequences for the entire archipelago. If the rainfall deficit is more than 50% over this critical period in one year, an outbreak can occur during February and March of the following year [16].

### 8.2. Availability of Larval Host Plants and Adult Host Fruits

Seasonality does not only influence the life cycle of *Eudocima phalonia*. Temperature, rainfall and relative humidity allow the rapid growth of *E. phalonia* larval host plants [7,16]. Because the survival rate of young larvae is primarily linked to the presence of young host plant leaves, strong vegetative growth is a favorable factor for larval development, whereas this is not a limiting factor for the more advanced larval instars. In the majority of cases, a long period of drought followed by intense rains can initiate vigorous growth of larval host plants [8]. In Australia, moth abundance is a dependent variable on the growth of plants, and larval host plant defoliation appears to limit *E. phalonia* reproduction during the dry season [27,54]. In Thailand, declining rainfall reduces the availability of larval host plants and decreases moth population abundance [8]. In Australia, the availability of larval host plants can be temporally separated, because if the main host plant is not available in sufficient quantity and quality during the optimal season of *E. phalonia*, then the pest can switch to other host plants, as is only observed in the Pacific Region on *Erythrina* trees [27,28,33].

Concerning the availability of adults’ host fruits, the favorable period for *E. phalonia* is in part due to the fruiting season in Malaysia [30,31], and in most cases, the peak of moth activity occurs when fruit maturity is approaching or near the first fruit harvest [7,8,16,28,60,61,133]. Despite acidity and low sugar in immature fruit, the number of pierced green fruit is higher at the beginning of the fruiting season, eight weeks before harvest, whereas at the end of the season, 77.6% of the attacks are on ripe fruits [28]. In fact, damages to green and acidic fruits are important only if moth density is very high, such as during outbreaks and if few ripe fruits are available [16,28,59,60,61]. For this reason, the first harvest can be seriously impacted even if fruits are still unripe. In addition, small orchards close to breeding sites experience much more damage than large ones which are distant from breeding sites [28].

### 8.3. Interhabitat Movement of Population

In New Caledonia, moth populations seem to have a density-dependent trigger to leave the breeding sites for feeding by progressive waves of interhabitat movement from mountain to plain biotopes in February [16,55]. The mountain biotopes are poorer in fruit than the plain ones, with mainly wild and small figs in the environment, whereas plains are more cultivated, with orchards offering some larger fruit areas for moths. Due to secondary larval host plants (*Erythrina fusca*) being widely present and close to orchards, the interhabitat movement of moth populations is quickly followed by egg-laying. This availability of larval host plants around orchards does not limit at all the development of *Eudocima phalonia* larvae and thus enhances population growth. As population density increases, moths disperse and then move across the plains in search of host fruits. Once the harvest period is over, host fruits are no longer available and moths come back to mountain biotopes, where they find wild or late fruits. In the same way, the lack of primary larval host plant in one environment may lead adult populations to seek more available egg-laying sites [27,28]. It is therefore mainly the availability of food and the egg-laying site that appear to be the two prevalent factors of population movement in *E. phalonia* [134]. When adult populations are high in New Caledonian orchards, larval outbreaks can be visible with *Er. fusca* defoliation. This can be mostly explained by the short distances between orchards and egg-laying sites [81]. However, this is not the case in Australia, where moths move between the breeding site and fruit orchards, covering great distances [135]. Population movements of *E. salaminia* and *E. phalonia* are also cited in order to overwinter in suitable localities between Queensland and New South Wales [73,136], allowing the reproductive period to be extended and the population abundance to be maintained. Hence, the phenology of the moths is modulated by food availability (larvae and imagines) and seasonal variability in climate. As in New Caledonia, small Australian populations of moths then remain in remote areas, which are far from orchards. Thanks to interhabitat movements, small populations are preserved in these favorable biotopes and allow the species to remain all year round, until the climatic conditions are more favorable in other biotopes.

### 8.4. Biocontrol Agent Complex Imbalance

In New Caledonia, a parasitism imbalance between mountain and lowland environments can be observed [16,90]. In mountainous areas, moth populations are limited by the stable rate of parasitoids and predators on eggs [81]. This parasitoid complex is therefore active in this biotope and can even be maintained during the dry season. Nevertheless, some outbreaks can be locally observed when parasitoids are absent. This is what happens when moth populations move to the plains and colonize new areas where parasitoid and predator communities are reduced or absent. Because the host spectrum of the main parasitoids is quite broad, *E. phalonia* is not the only host and alternative species maintain small parasitoid populations. In addition, the change in oviposition behavior (solitary or egg cluster) and the high reproductive capacity of the female does not allow for all parasitoids to attack eggs, even if some of them, such as *Telenomus* or *Trichogramma*, have a preference for eggs laid in clusters, whereas *Ooencyrtus papilionis* shows a preference for solitary eggs. High density of eggs, young leaves of *Erythrinae* spp. and the decline in the parasitism and predation rates can strongly favor an outbreak of larvae and, consequently, a proliferation of an adult population. Furthermore, the moth life cycle is also faster than that of the parasitoids and predators and their populations cannot immediately follow the expansion of *E. phalonia* and this creates a lag between the pest population peaks and the parasitic/predatory complex [90]. This contributes greatly to an increase in the pest’s prevalence. In Australia, the same phenomenon is observed during the wet season and parasitoid activity is also insufficient to control moth populations [74]. However, no larval outbreak can be observed there as seen in New Caledonia, mainly because larval host plant and adult host fruit areas are more distant from each other.

Recovery at a low population level is slow and occurs when populations of parasitoids and predators increase, when the high availability of their prey helps to exercise control over the populations of *E. phalonia*. Predators such as birds and wasps change their behavior and the rate of predation increases by consuming or destroying many larvae [16]. However, for several years, smaller and scarcer *Polistes olivaceus* nests have been observed. Climate change (including recurrent droughts) and bush fires have probably contributed to the decrease in these wasps. Moreover, these recurring events could be responsible for the decrease in prey (caterpillars and pupae), making it impossible for the colonies to build enough founders from year to year and therefore the populations are diminishing [50]. At the same time, the overcrowding of larvae leads to intense competition for access to foliar resources. Larval mortality is increasing and stressed larvae are dying [16] or producing smaller and less fertile moths, causing the decline of the *E. phalonia* population [88]. One can easily assume that in nature, dwindling resources can have the same consequences for the pest population.

## 9. Pest Management

### 9.1. Mechanical and Physical Means

While hand capture and manual destruction of moths are cited by many authors [32,55,63,107], this is an inefficient method, especially during outbreaks. It involves a continuous nocturnal presence and increased observation in orchards under light. On large cultivated areas, this method is not conceivable and the catching effort is huge compared to the results obtained. Individual fruit bagging seems rather effective [26]. However, this largely depends on the material studied by the different authors—for example, polyethylene bags or insect nets have the best results and prospects but these two materials promote fruit rot or maturity delay [137,138]. In addition, the method is time-consuming and expensive because it involves the individual protection of all fruits for each tree [60]. The method, however, may be applicable on a small scale or if the crop is of high value [60,76].

On a larger scale, the option of nylon nets extended on each orchard line or by tree remains a possible alternative but in the short term. It could help to protect crops from other pests too (birds, fruit bats or fruit flies) if the stitch size is adapted [16,60,61,139]. For *Eudocima phalonia*, a 2.5-cm mesh already protects 80% of the fruits [139] while a minimum 1-cm mesh is recommended by several authors [16,60,138]. This method met with great success in Australia and in American Samoa against *E. materna* [139] and a number of secondary fruit-piercing pest moths in Japan [137]. Spraying a contact insecticide on the net surface was also recommended [76]. While protective nets have some advantages for small areas or isolated trees [82], they require a considerable investment even if they can be used for several years. In New Caledonia, a local fruit farmer with a 17-hectare orchard would need to invest 25 million Pacific Francs (more than USD 236,000), including a special machine to install them, meaning more than 1,400,000 Pacific Francs (USD 14,200) for the protection of a single hectare [140]. Furthermore, structures must be completely disassembled during cyclonic events (including winds and floods) and then reassembled once the alert is lifted. Besides this, it is necessary to avoid the weight of the net on trees, which can be destructive. Other local fruit farmers also reported an early or late ripening of fruits and advanced defoliation of fruit trees [88]. Notwithstanding these significant disadvantages, nylon nets are currently a practical and effective method, but they are neither cheap nor quick to implement and cannot be used in the long term.

### 9.2. Fruit Crop Management

The modification of the cultivation pattern is recommended by many authors with different points of view. The planting pattern, period and method of harvest, as well as the cultivated cultivars, can be modified according to the flight periods of *E. phalonia* populations. Some authors note that the peripheral lines of orchards are attacked first and then along planting lines and so propose to plant the trees in a compact scheme in order to reduce *E. phalonia* impacts [27,60,107]. However, no study has yet quantified the effectiveness of this scheme and this method may possibly be applied only to young orchards, shortly after establishment. Similarly, some fruit species and cultivars appear to be more or less attractive than others [27,39]. In this case, the strategy consists of using attractive plantations such as a trap essence near orchards or by suspending more attractive fruits able to avoid piercing on high-economic-value crops [38]. Fallen, rotting or pricked fruits, on the tree or on the ground, need also to be collected and destroyed, because their maceration can create additional attractants for primary fruit-piercing moths [27,28,141] and secondary fruit-piercing or fruit-sucking moths [107].

To avoid damages by *Eudocima phalonia*, other authors suggest selecting fruit species and cultivars with fruiting and ripening periods outside of the ripening season [28,38,60,81]. In the same way, the first harvest can be aborted in favor of the second fruiting season (outside the *E. phalonia* season) when species and cultivars are biennial, but this reduces tree productivity and profitability for producers since only one harvest is carried out instead of two [7]. If this method is not suitable for farmers, regulating fruit maturation or early harvest when the first signs of fruit maturity are observed in order to minimize damages have little impact on production [28]. This type of harvest management has provided satisfactory results in Africa and reduced damage from 93% to 10.7% and from 98.5% to 23% for grapes and grapefruits, respectively, for various species of fruit-piercing moths [39]. However, fruits of conventional size and which are still green need to be force-ripened after harvest [28], which must certainly have an impact on the taste and nutritional quality as well as on the cost of fruit production. Likewise, the method is not applicable to all fruit species and cultivars (such as mangoes or papayas) that are climacteric, needing some time on trees to ripen [27] and for some crops, the harvest period is not adaptable.

### 9.3. Larval Host Plant Suppression

According to the literature, suppression of larval host plant is by far the most recommended and cited method [16,55,76,94] but its effectiveness can be questioned, especially in the New Caledonian context. Indeed, in New Caledonia, the presence of secondary larval host plants (*Erythrina fusca* and *Er. variegata*) makes it more difficult to suppress larval host plants in the environment due to their wide distribution, as they were planted to provide some shade for the coffee plantings several decades ago. Only one reference mentioned the effect of larval host plant suppression in New Caledonia [55]. Before the outbreak of 1969, almost 2000 *Er. fusca* trees near citrus orchards were cut down or destroyed in a mountain valley. While losses were close to 100% in most of the territory, damage was reduced to 42% in the study valley on tangerines. In the Loyalty Islands, *E. phalonia* attacks are annual and an attempt to destroy *Er. fusca* trees has been proposed on the islands of Lifou and Maré [55] but the results have not been reported. Even if some *Erythrina* species are currently heavily affected and have been declining over the last 10 years due to the Erythrina Gall Wasp, *Quadrastichus erythrinae* (Kim) (Hymenoptera, Eulophidae), Menispermaceae are numerous in the primary forests and *E. phalonia* develops without difficulty on *Er. fusca*. Thus, suppression of these plentiful trees appears impossible given the cost of a campaign to destroy these massive trees [16,55]. In addition, they are also used in the Kanak pharmacopoeia [142]. Despite being able to eradicate these larval host plants, a heterogeneity pattern in the landscape could eventually decrease the impact of moths and maintain predatory and parasitic fauna [16]. However, interhabitat movement of moth populations should not be neglected, as they are able to fly over 25 km [15,16,134]. Destruction of larval host plants is more likely a preventive measure than an effective means of control, especially during the peak moth population period [18]. Although the technique seems possible and effective locally or on small islands, it is difficult to implement in most large territories.

### 9.4. Chemical Control with Synthetic and Natural Substances

Organochlorines or organophosphates, DDT (Dichlorodiphenyltrichloroethane), flubendiamide, arsenates or naphthalene were used against *Eudocima phalonia* adults before being prohibited in agriculture due to their toxicity for the environment, users and consumers. Their use is highly controversial as it has limited effectiveness [30,76,137] and because spreading insecticides is judged ineffective. The moths spend little time on a fruit, they are not sedentary and are often absent at the time of treatment and consume only the juice. The amount of contact and absorption of insecticides are therefore insufficient for such a large insect [31,55,138]. In addition, moths attack fruits close to maturity and therefore to harvest and their marketing, so the use of chemicals at this stage of maturity jeopardizes food safety due to contamination with persistent insecticide compounds [27,55,107,138]. Therefore, the only chemical control of the pest focuses on larvae and their host plants. For example, if novaluron and lufenuron have a limited action on *E. materna* larvae, flubendiamide (anthranilics) is very effective [108]. Furthermore, in a larger sense, the use of broad-spectrum insecticides is no longer appreciated by both the consumers and the farmers. Instead of chemical substances, natural active substances can be promoted for their insecticidal effect or growth inhibition against some Lepidoptera larvae [143,144,145]. The best possibilities are molecules of the alkaloid family, also known for their repellent, palatable and insecticidal effects on several insects [144,146,147,148,149,150]. If such substances have been proposed as control means against *E. phalonia*, it should not be forgotten that the larval host plants (Menispermaceae and Erythrinae) naturally contain some alkaloids, and the use of this type of molecule could be ineffective in the long term [15]. For example, *Oraesia excavata* (Bütler) (Lepidoptera, Erebidae) is insensitive to the feeding inhibitory or the insecticidal effect of isoboldine and cocculolidine, because the species develops on Menispermaceae [146]. Therefore, these natural bioactive substances would always have to be applied as a mix instead of a single chemical extracted from the plants, to avoid any tolerance or resistance. Finally, the use of bacteria, such as *Bacillus thuringiensis* (Berliner) (Bacillaceae), has become a common practice since the 1920s and 1930s in biological control, particularly against Lepidopteran larvae, including those of *E. materna* [108,151] and many pests around the world [152]. Although it has some advantages, many disadvantages are beginning to be pointed out by several studies. Because of its broad spectrum of effectiveness against insect target species, it can have an indirect impact on the parasitoids associated with them [3,153]. Moreover, the widespread use of *B. thuringiensis* is gradually leading to resistance [154,155,156,157]. Other bacterial pathogens are also available, such as *Saccharopolyspora spinosa* (Mertz and Yao) (Pseudonocardiaceae) (widespread) or *Photorhabdus luminescens* (Thomas and Poinar) (Enterobacteriaceae), cited as active on *E. materna* larvae, and provide perspectives for biological control against the genus *Eudocima* [108,151]. On other Lepidoptera models such as *Helicoverpa zea* (Boddie) (Lepidoptera, Noctuidae), the toxin produced by *Sa. spinosa*, can even be coupled with a food attractant [158]. Whether chemical or natural, and given the ecological niche of *E. phalonia* larvae in New Caledonia, spraying such substances into the natural environment is almost impossible and unaffordable, according to the endemicity and diversity of other species, potentially threatened or eradicated by this method [7,31,82]. Nevertheless, natural substances with insecticidal activity should be explored and can be used in order to develop poisonous fruit baits for adult moths. Although no scientific reference discusses *Sa. spinosa* as adult moth insecticide, an evaluation of one of its formulations (Success^TM^) on *E. phalonia* has given good results in laboratory and partial field conditions [88]. However, to avoid any resistance phenomena, these types of substances could be used alternately or easily replaced by another natural substance with insecticidal activity or used in rotation.

### 9.5. Light Control

The contradictory results raised by numerous observations indicate that the wavelength emitted by the lamps can either attract or repel depending on the phototropism of the noctuid species [82,107,159,160]. This method of illumination to either attract individuals or disrupt their behavior nevertheless requires facilities and a substantial financial investment. Repulsive lights have wavelengths between 550 and 580 nm [25,107,137,161,162,163,164]. Fluorescent lamps and mercury vapor lamps are among the most cited in the literature [107,133] but incandescent lamps (yellow) including LED lamps are good alternatives in terms of financial cost, energy and resistance and are able to emit a precise wavelength for target insects [133,165,166]. Intense bilateral or total orchard brightness gives excellent results in reducing the damages of several fruit-piercing and fruit-sucking moths [21,114,133,161,163,167]. The resulting repulsion effect is, in fact, a resting and immobilization behavior during the daytime phase [107]. Although the illumination of the orchards prevents *Eudocima phalonia* from piercing the fruits, it could be combined with manual capture and destruction of individuals when they are nearby [15]. However, if moths in the illuminated area are in resting position and avoiding fruits, it does not control reproduction and egg-laying on the larval hosts that can take place elsewhere.

On the other hand, although it is well known in the literature that light traps are often used in moth inventory methods because UV lights (or black lights) are considered attractive to nocturnal insects [166], the method failed for *Eudocima phalonia* in various places [7,63,138]. In New Caledonia and in Australia, these lights have yielded no conclusive results [122,163]. Moreover, few specimens of *E. phalonia* are observed revolving around the exterior lights of dwellings or in private homes. If a light wavelength effect can be observed on *E. phalonia*, illuminating the orchards presents an additional control method and should be quantified. Globally, the energy required for this method remains disproportionate and is not always accessible in terms of field logistics and also increases the price of consumables [16,107,163]. Nevertheless, this method, using attractivity or repellency, should be investigated more deeply in the control of fruit-piercing moths, especially with the advent of LED lights, which became economically accessible and which require much less energy than conventional electric bulbs. Moreover, the advent of solar energy represents an affordable and autonomous power source in the fields.

### 9.6. Introduction of Parasitoids and Predators for Population Control

Although parasitoids and predators are identified for *Eudocima phalonia*, it was previously seen that according to locality and species, their incidence and parasitism or predation rates have limited effectiveness in the environment. Larval parasitoids cited in the literature for biological control include the genera *Ooencyrtus*, *Trichogramma* and *Telenomus*. One of the species, *Ooencyrtus* sp. (*papilionis* group), was successfully released and established in the Cook Islands, Fiji Islands and Tonga but not in Samoa [116,118]. In New Caledonia, *O. papilionis* occurs naturally but is not specific to *E. phalonia* and can, for instance, parasitize eggs of *Papilio montrouzieri*, an endemic Papilionidae [81]. Other species, as *O. crassulus*, were introduced in Tonga and the Cook Islands and the egg parasitism rate has since increased and declining moth populations were observed, especially in the Cook Islands [116,118,168]. *Telenomus lucullus* is established in Papua New Guinea and was successfully introduced in Tonga, Fiji Islands, Samoa and showed some good results [116,118,168]. These parasitoids seem to be potential candidates for biological control because they are more specific, preferentially attack egg clusters and can have a high parasitism rate during outbreaks. Finally, the genus *Trichogramma*, e.g., *T. ostriniae*, shows a 95% parasitism rate for the eggs of *E. phalonia* in Hawaii [117], whereas *T. chilonis* does not appear to control the populations. In New Caledonia, this larval parasitoid has only an insufficient impact [81]. Globally cited parasitoids are not specific to *E. phalonia* and may have an impact on the rich endemic insect fauna of New Caledonia. An investigation of the biogeographic origin of this pest species could help to identify some more specific parasitoids which could be used in areas where it has spread.

Concerning larval parasitoids, their application is limited to the tachinid fly *Winthemia caledoniae* (Diptera, Tachinidae) or from wasps of the genus *Euplectrus* (Hymenoptera, Eulophidae). Although introductions of *W. caledoniae* were attempted in Tonga and in the Fiji Islands, in 1987 and 1983, respectively, such species were not established in the two archipelagos [59,118,169]. In New Caledonia, this endemic fly controls moth populations only at the end of the population peak [16]. The best parasitoid species appears to be *Euplectrus maternus* because of its ability to parasitize other species of *Eudocima* in the laboratory [119]. Despite encouraging laboratory results, its introduction into Guam was not successful [79]. A second species, *Eu. melanocephalus*, appears to be specific to the *Eudocima* species but its potential introduction into other Pacific islands requires further studies [116]. Hymenoptera are nevertheless good candidates for biological control, particularly in Australia, but their activity during the wet season is generally insufficient to maintain the pest population below the threshold of economic damages [33,121].

While egg or larval parasitoids are widely cited for biological control, the introduction of predators is less common and must be previously and carefully studied in the laboratory. Few observations are mentioned but a predatory bug, *Podisus maculiventris* (Say) (Hemiptera, Pentatomidae), can feed on *Eudocima phalonia* larvae but not only on this species [170]. It is therefore important to assess the impact on target and non-target species that may be affected before the introduction of a predator or parasitoid and their potential action during outbreaks. In general, the introduction of parasitoids or predators should always be assessed in the most appropriate manner to avoid creating an imbalance of species in the environment. Indeed, the introduction of parasitoids in the case of *E. phalonia* could probably allow control of the population, but it could also impact the other species of *Eudocima* whose behavior is quite different. In New Caledonia, *E. salaminia* and *E. materna* are currently not problematic and could suffer a significant impact by reducing the populations size until it disappears. In other Pacific islands, where some native *Eudocima* species are found in sympatry with *E phalonia*, addressing the impact of the introduction of parasitoids could certainly affect these local communities [47]. Such an option must involve a rigorous risk analysis to avoid any impact on these native communities.

### 9.7. Chemical Ecology: Attraction or Repellent Phenomena

#### 9.7.1. Repellent Substances

While repellency may show some control prospects, it is a phenomenon sometimes camouflaged by the neutralization of odors. Neutralizing an odor and repelling an insect are two different mechanisms, one is a lack of recognition of olfactory molecules and the other is the recognition of an unpleasant or repulsive molecule. While no repellent molecules have yet been identified for *Eudocima phalonia*, β-styryl butyl ketone (or 4-methyl-1-phenylhex-1-en-3-one) can repel *Oraesia excavata* and *E. tyrannus* and reduces the damages of these two species in peach orchards in Japan [171]. The use of non-specific essential oils is also cited in the literature as a repellent. Against *E. materna*, citronella essential oil (*Cymbopogon citratus*, Poaceae) showed a repellent effect in the laboratory, as well as the essential oils of *Cascabela thevetia* (Apocynaceae), *Papaver argemone* (Papaveraceae), *Jatropha curcas* (Euphorbiaceae) and *Millettia pinnata* (=*Pongamia glabra*, Fabaceae) [172]. More generally, essential oils are reported to have repellent activity on some insect species [173]. In the agroforestery systems in Brazil, freshly cut branches and trunks of the Lemon Scented Eucalyptus *Corymbia citriodora* (Myrtaceae) are used as repellents against moth species [174]. Indeed, the essential oil of *C. citriodora* is cited to be attractive at low concentrations and supposed to have a repellent activity at high concentrations against some moth insect pests [175]. This plant occurs in New Caledonia and may be of prospective use if more studies are carried out to explore its effects on *E. phalonia*. The neem oil (*Melia azedarach*, Meliaceae), already known as an insecticide, is able to repel moths such as *E. materna* in the laboratory but not in the field [172]. Although some repellent activity can be found with essential oils [173], they can, however, be phytotoxic if they are directly sprayed on plants [176]. Therefore, their use needs to be further investigated.

#### 9.7.2. Neutralizing Substances

Concerning neutralizing odors, oil emulsion is recommended for integrated pest management (IPM) in various insect pest models [30,76,177]. In Malaysia, mineral oils (at 0.35%) were sprayed weekly until the fruits were ripe and a decrease in the damages caused by *Eudocima phalonia* was recorded in orange orchards [30]. However, the oil emulsion has to be sprayed every 10 days to maintain the deterring effect [76]. Direct fumigation in the orchards can also be used to mask the smell of fruits and keep away nocturnal insects while the smoke is present, by burning oil and various plant materials [32]. This appears to be quite effective [76] but the method is constrained by climatic conditions (wind, rain), which can sometimes seriously reduce its efficiency. In addition, it is effective for only one night and must therefore be repeated every night during the fruiting season [32] and moths return to orchards as soon as the smoke dissipates. The release of smoke from different types of substances can also contribute to the deterioration of the surrounding air quality.

#### 9.7.3. Attractive Substances

Attraction phenomena primarily consist of locomotory behavior towards a source triggered by the physiological recognition via an antennal response to some molecules contained in a plume odor. Unlike repulsion methods, attraction has long been used in different ways to divert pests from attack-sensitive crops with the use of attractive plantations such as a trap crop or by suspending attractive fruits to protect a part of the crop. This is the case for ripe tomatoes, bananas or guavas, which seem to prevent piercing on oranges until these trap crops are no longer available around orchards. In this way, damages are reduced on oranges and moths are concentrated around trap crops [31,58,76]. However, once the resource is depleted, moths attack other fruits. Coupling these attractive fruit baits with different insecticides can avoid damages and potentially reduce moth populations [39]. A food bait based on these attractive fruits and sugar (or molasses), in which an insecticide is incorporated, was, for example, effective against *Eudocima materna* in India [172]. Several authors have therefore tested different food baits containing various dosages, types of sugars and additives. A South African-formulated bait based on brown sugar and sodium arsenate appears to be effective in attracting various moths including *E. phalonia* but primarily in laboratory conditions [7]. The use of sweet molasses bait in which 50% malathion (organophosphates) is added can be recommended by some authors [76]. In India, a mixture of water, raw sugar, fruit pulp and arsenate was used as an attractive solution (replaced weekly) [32]. Other solutions containing beer, vinegar, alcohol or unrefined sugar are also cited in the literature [7,160]. However, depending on the chemo-physical features of insecticidal substances, especially when these are on the bait surface, detection by moths can easily be supposed, and they can push them away from poisoned fruits. The best option for an attractive substance to *E. phalonia* is to arrange a baiting mixture containing ester, aldehyde and alcohol components coupled with sugar [162,163], along with an insecticidal substance eliciting no olfactory response, as a “Lure and Kill strategy”. Recently, some sex pheromone components involved in mate recognition were identified in *E. materna* [178]. Depending on the mixture formulation from different ratios of compounds, a blend containing (Z,E)-9, 11-Tetradecadienyl acetate, Z-9-Tricosene, Z-9-Pentacosene and 2-Ethyl Hexanol was the most effective in field trapping experiments, with a possible synergism between the pheromone components of *E. materna* and the 2-Ethyl Hexanol compound [178]. This recent study provides new prospects for the possible existence of a sex pheromone in *E. phalonia* and its potential use in IPM.

## 10. Conclusions

The review of current pest management strategies against *Eudocima phalonia* points out that while some of these strategies may have a positive effect in small orchards, they present many disadvantages on larger scales. In the New Caledonian context, the population dynamics of the species have evolved over the past forty years and recent observations have shown that the favorable period for optimal development tends to extend during the first half of the year, with more outbreaks. The extent of the favorable period is probably linked to the multiple effects of climate and environmental changes, especially for outbreaks. As a result, the common fruit-piercing moth is becoming a serious threat in New Caledonia, but also in other parts of the world where the species is encountered. The preservation of resources, food self-sufficiency and food security are the keys to the future of each country and, as such, they deserve to be addressed with innovative, sustainable and eco-friendly solutions. Of course, we must balance dealing with a pest and preventing the erosion of biodiversity. In the dynamics of an agro-ecological transition, new, original research prospects need to be further studied, such as aspects of chemical ecology. Because amongst all solutions proposed by several authors, many of them lack specificity and could have an impact on the environment or on beneficial insects, the use of attractive kairomones or specific pheromones (already effective for many other insect pests) seems to be a promising path to explore to prevent fruit crop damages. However, the chemical ecology of the moth has not been so well investigated, although recent studies have helped to identify potential pheromone compounds involved in mate recognition of *E. materna* and, by extension, probably in *E. phalonia*. Electrophysiological and behavioral studies are needed and further progress in this area should be encouraged in order to find an effective solution against this pest, not only in New Caledonia but also elsewhere.

## Figures and Tables

**Table 1:** Assessment of damage to fruit species for all species of fruit-piercing moth, clearly quoting or including *Eudocima phalonia*. Damage is defined as a measurable loss of commodity value in terms of quantity, quality or aesthetic appeal of fruits, due to insect activity.

Fruit Species	Country	Damage	Reference
	Africa		
All fruit species	Pacific islands	<80%	[16,38,62,63]
	Sri Lanka		
Carambola (*Averrhoa carambola*)	Australia	<50%	[64]
Citrus	Australia	<80%	[64]
(All cultivars, *Citrus* sp.)	China	<50%	[65]
	Fiji islands	10–15%	[59]
		73%	[66]
	India	10–55%	[67]
	Malaysia	3–5%	[68]
		5–90%	[30]
		17–39%	[30]
Grapefruit (*Citrus paradisi*)	Thailand	20–36%	[8]
	India	4–90%	[69,70,71,72]
Mandarin (*Citrus reticulata*)	New Caledonia	70–100%	[15,55]
	Thailand	11–18%	[8]
Orange (*Citrus sinensis*)	Australia	25%	[73]
	India	>50%	[28,74]
		30–40%	[70]
	Sri Lanka	10–55%	[67,70]
		21–42%	[32]
Grape (*Vitis* sp.)	South Korea	8.9%	[75]
Lychee (*Litchi chinensis*)	Australia	<50%	[64]
Pear (*Pyrus* sp.)	South Korea	3.4%	[75]
Pomegranate (*Punica granatum*)	South Korea	20–40%	[58,76]
Tomato (*Punica lycopersicum*)	Samoa	100% (outbreak)	[14,18]

**Table 2:** List of fruit species and cultivars pierced by *Eudocima phalonia* in New Caledonia (for the botanical classification, references are found on www.catalogueoflife.org and www.tropicos.org). GDSV: *Groupement de Défense Sanitaire Végétal*, Plant Health Defense Group.

Scientific Name	Reference
ANACARDIACEAE	
*Mangifera indica* (Mango)	[14,16,82,83,84,85,86,87,88]
*Spondias dulcis* (Golden apple, Ambarella, Polynesian plum)	[85,89]
ANNONACEAE	
*Annona* sp.	[55]
*Annona muricata* (Soursop)	[16,82,83,85]
*Annona reticulata* (Custard apple)	[55]
*Annona squamosa* (Sugar apple)	[16,83,85]
BROMELIACEAE	
*Ananas comosus* (Pineapple)	[14,82,83]
CARICACEAE	
*Carica papaya* (Papaya)	[16,81,82,83,84,85,86]
CUCURBITACEAE	
*Cucumis melo* (Melon)	[15,16,55,83,90]
*Citrullus lanatus* = *C. vulgaris* (Watermelon)	[16]
ELAEOCARPACEAE	
*Elaeocarpus* sp. (Shiva’s tear)	[14]
LAURACEAE	
*Persea* sp. (Avocado)	[91]
MORACEAE	
*Ficus* sp. (Wild fig) *Ficus carica* (Fig)	[14,16,89,90]
MUSACEAE	
*Musa* sp. *Musa x paradisiaca* = *Musa sapientum* (Banana) Plantain cultivars	[16,82,83,92]
MYRTACEAE	
*Psidium* sp. i.e., *Psidium guajava* (Guava)	[14,16,82,83,85,86,87,88,89,90]
*Syzygium cumini* = *Eugenia jambolana* (Black plum)	[14,16,83,85,86,89]
*Syzygium jambos* = *Eugenia jambosa* (Pink-apple)	[90]
OXALIDACEAE	
*Averrhoa carambola* (Carambola)	[16,85,86,87,88]
PASSIFLORACEAE	
*Passiflora edulis* (Passion fruit)	[86,91]
ROSACEAE	
*Fragaria* sp. (Strawberry)	[93]
*Prunus persica* (Peach)	[16,85,86]
RUBIACEAE	
*Coffea* sp. (Coffee)	[82,83,92]
*Coffea arabica* (Arabian coffee)	[16,83,92]
RUTACEAE	
*Citrus* sp.	[55,85,86,87,88,89,90,94]
*Citrus aurantiifolia* (Key lime)	[85,86,91]
*Citrus grandis* = *Citrus maxima* = *Citrus paradisi* (Grapefruit)	[16,85,86,88,93]
*Citrus latifolia* (Tahitian Lime)	[86,88,91,93]
*Citrus limon* = C. *medica var. limon* = *C. limonum* (Lemon)	[16,88,92]
*Citrus nobilis* = *C. aurantium = C. tangelo* (Bitter orange)	[16,85,86,87,88,92,93]
*Citrus reticulata* (Mandarin) including all cultivars	[16,55,82,83,85,86,87,88,93]
*Citrus sinensis* (Sweet orange) including all cultivars	[16,55,83,85,86,87,88,90,91,93,95]
SAPINDACEAE	
*Litchi chinensis* (Lychee)	[16,85,86,87]
SOLANACEAE	
*Solanum lycopersicum* (Tomato)	[16,55,81,82,83,90]
VITACEAE	
*Vitis* spp. *Vitis vinifera* (Grape vine)	[82,83]

**Table 3:** Life cycle of *Eudocima phalonia* according to localities and different authors.

Country	Host Plant	Egg	Larva	Chrysalis	Total	Reference
Malaysia	*Leea indica* (Vigna)	3 ± 1 d	20.8 ± 1 d	15.8 ± 0.5 d	39.6 ± 2.2 d	[31]
Guam	*Cocculus* sp. (Menispermaceae)	-	-	-	24.3 ± 0.75 d	[24]
	*Erythrina variegata* (Fabaceae)	-	-	-	23.2 ± 1.02 d	[24]
	*Erythrina variegata* (Fabaceae)	-	25.1 ± 8.7 d	-	-	[102]
	*Tinospora homosepala* (Menispermaceae)	-	22.2 ± 0.8 d	-	-	[102]
Fiji Islands	*Erythrina variegata* *Er. lithosperma* (Fabaceae)	4 d	14–28 d	17–21 d	43 d	[59]
India	Not specified	3–4 d	14–16 d	13–14 d	30–34 d	[109]
	*Tiliacora* sp. (Menispermaceae)	3–4 d	13–17 d	12–18 d	28–39 d	[110]
	*Tiliacora* sp. (Menispermaceae)	3–4 d	15 d	21 d	39–40 d	[111]
	*Tiliacora* sp. (Menispermaceae)	14 d	28 d	15 d	57 d	[112]
India	*Tiliacora* sp. (Menispermaceae)	3–4 d	13–15 d	8–10 d	24–29 d	[70]
Sierra Leone	Not specified	3 d	13–20 d	10–14 d	26–37 d	[7]
Sri Lanka	*Anamirta* sp. (Menispermaceae)	2–3 d	17–20 d	14–16 d	-	[113]

## Data Availability

Not applicable.
